# Long Non-Coding RNA Hotairm1 Promotes S100A9 Support of MDSC Expansion during Sepsis

**Published:** 2020-09-22

**Authors:** Tuqa Alkhateeb, Isatou Bah, Ajinkya Kumbhare, Dima Youssef, Zhi Q Yao, Charles E McCall, Mohamed El Gazzar

**Affiliations:** 1 Department of Internal Medicine, East Tennessee State University College of Medicine, Johnson City, TN 37614, USA; 2 Department of Internal Medicine, Section of Molecular Medicine, Wake Forest University School of Medicine, Winston-Salem, NC 27157, USA

**Keywords:** Myeloid-derived suppressor cells (MDSCs), Sepsis, Immune response, Inflammation, Mice

## Abstract

Myeloid-derived suppressor cells (MDSCs) expand during mouse and human sepsis, but the mechanism responsible for this is unclear. We previously reported that nuclear transport of S100A9 protein programs Gr1^+^CD11b^+^ myeloid precursors into MDSCs in septic mice. Here, we show that long non-coding RNA Hotairm1 converts MDSCs from an activator to a repressor state. Mechanistically, increased Hotairm1 expression in MDSCs in mice converted S100A9 from a secreted proinflammatory mediator to an immune repressor by binding to and shuttling it from cytosol to nucleus during late sepsis. High Hotairm1 levels were detected in exosomes shed from MDSCs from late septic mice. These exosomes inhibited lipopolysaccharide-stimulated secretion of S100A9 from early sepsis Gr1^+^CD11b^+^ cells. Importantly, Hotairm1 knockdown in late sepsis Gr1^+^CD11b^+^ MDSCs prevented S100A9 cytosol to nuclear transfer and decreased repression of proimmune T cells. Notably, ectopic expression of Hotairm1 in early sepsis Gr1^+^CD11b^+^ cells shuttled S100A9 to the nucleus and promoted the MDSC repressor phenotype. In support of translating the mechanistic concept to human sepsis, we found that Hotairm1 binds S100A9 protein in CD33^+^CD11b^+^HLA-DR^−^ MDSCs during established sepsis. Together, these data support that Hotairm1 is a plausible molecular target for treating late sepsis immune suppression in humans and its immune repressor mechanism may be cell autonomous.

## INTRODUCTION

Life threatening sepsis dysregulates the host response to uncontrolled infection [1]. The immune response to sepsis enhances myelopoiesis to compensate for innate immune cell loss during excessive inflammation [2,3]. However, progenitors and precursors of monocyte, neutrophil and dendritic cells fail to differentiate into competent immune cells and may be reprogrammed into immunosuppressive phenotypes [2,4,5]. These myeloid-derived suppressor cells (MDSCs) suppress the innate and adaptive immune responses and thus hamper inflammation resolution and immune homeostasis [4-7]. MDSC numbers increase in blood in mice with sepsis [2,4,8], and are found in septic patients that may become chronic (defined as having sepsis for 14 days or longer) and develop critical illness [7,9]. The molecular mechanisms underlying MDSC development in sepsis remain unclear.

Sepsis is initiated by an early/acute proinflammatory reaction, which may be rapidly lethal, or progress to a late/protracted immunosuppressive stage characterized by decreases in competent innate and adaptive immune cells and continued increases in mortality [10-12]. We reported that S100A9 contributes to postsepsis protracted immunosuppressive state [13]. S100A9 protein is a common constituent of myeloid-derived circulating leukocytes and amplifies inflammation [14,15]. It is induced in myeloid cells by inflammation-derived signals [14,16,17]. We found that MDSCs expansion decreases in S100A9-deficient mice during polymicrobial sepsis. S100A9 plasma protein levels increase in acutely septic mice, but decrease in mice with protracted immunosuppression. Gr1^+^CD11b^+^ myeloid cells generated during acute sepsis are proinflammatory [4]. Notably, Gr1^+^CD11b^+^ myeloid cells generated during chronic sepsis (i.e., MDSCs) fail to secrete S100A9 upon ex vivo stimulation with bacterial lipopolysaccharide [13]. S100A9 localizes mainly in cytosol during acute sepsis, but moves to the nucleus as immunosuppressive Gr1^+^CD11b^+^ MDSCs emerge. Thus, S100A9 can contribute to the proinflammatory or anti-inflammatory states typical of sepsis.

The role of long non-coding RNAs (lncRNAs) in sepsis is unclear. LncRNAs contain >200 nucleotides, which like protein-coding mRNAs, are polyadenylated, capped and spliced [18,19]. Recent evidence indicates that lncRNAs control production of inflammatory mediators and cell fate [20,21], They are expressed in a cell- and tissue-specific manner [20], with differential expression during cell activation [21]. While most LncRNAs interact with protein, RNA, or DNA [22,23], immune-related lncRNAs prefer protein binding, protein modifications and transport [21,22,24]. In this study, we profiled expression of lncRNAs in Gr1^+^CD11b^+^ MDSCs during sepsis. Here, we found differential expression of myeloid-related lncRNA Hotairm1 in late sepsis. The results show that it controls MDSC repressor cell function by binding to and transferring S100A9 to the nucleus. Hotairm1 may provide a molecular targeting site for post-acute sepsis correction of immune suppression.

## MATERIALS AND METHODS

### Mice

Male C57BL/6 mice (8 to 10 weeks old) were purchased from the Jackson Laboratory (Bar Harbor, ME). The mice were housed in a pathogen-free facility, and were acclimated to the new environment for a week before surgery. All experiments were conducted in accordance with National Institutes of Health guidelines and were approved by the East Tennessee State University Animal Care and Use Committee.

### Polymicrobial sepsis model

Sepsis was induced by cecal ligation and puncture (CLP) in mice as described previously [25]. Briefly, a midline abdominal incision was made and the cecum was ligated distal to the ileocecal valve, and punctured twice with a 23-gauge needle. A small amount of feces was extruded into the abdominal cavity. The abdominal wall and skin were sutured in layers with 3-0 silk. Sham-operated mice were treated identically except that the cecum was neither ligated nor punctured. Mice received (i.p.) 1 ml of lactated Ringers plus 5% dextrose for fluid resuscitation. To establish intra-abdominal infection and approximate the clinical conditions of human sepsis where there is a delay between the onset of sepsis and the delivery of therapy [26], mice were treated (s.c) with antibiotic (imipenem; 25 mg/kg body weight) diluted in saline (0.9% sodium chloride) at 8 and 16 hr after CLP. The level of injury and manipulation create prolonged peritoneal infections with high mortality (~60-70%) during the late/chronic phase [25], as monitored for 28 days. Mice moribund during acute/early and chronic sepsis were euthanized and analyzed. For comparison purposes, we define early/acute sepsis as the first 5 days after CLP and late/chronic sepsis post day 6. A corresponding number of mice from the control/sham group were also analyzed at the same time points.

### Sepsis patients

Twenty-one patients 18 years of age or older who were admitted to Johnson City Medical Center and Franklin Woods Hospital in Johnson City, Tennessee, and who were diagnosed with sepsis or septic shock were included in the study. Criteria for patient selections were as described previously [13]. Patients were divided into two categories: early septic and late septic, relative to the day of sepsis diagnosis. The early/acute septic group included patients within 1-5 days of sepsis diagnosis. Those who have been septic for more than 6 days were considered late/chronic septic. For this latter group, blood was drawn at days 6-68 after sepsis diagnosis. Blood from healthy control subjects was supplied by Physicians Plasma Alliance (Gray, TN). The study was approved by the Institutional Review Board (IRB) of the East Tennessee State University (IRB#: 0714.6s). Signed informed consent was obtained from all participants.

### Cell isolation

Gr1^+^CD11b^+^ cells were isolated from bone marrow using magnetic beads according to the manufacturer's protocol (Miltenyi Biotech, Auburn, CA). Briefly, the bone marrow was flushed out of the femurs with RPMI-1640 medium (without serum) under aseptic conditions. A single cell suspension was made by pipetting up and down and filtering through a 70 μm nylon strainer, followed by incubation with erythrocyte lysis buffer and washing. The cell suspension was subjected to a positive selection of the Gr1^+^ cells by incubating with biotin-coupled mouse anti-Gr1 (Cat # 13-5931-85/Clone RB6-8C5; eBioscience, San Diego, CA) and anti-CD11b (Cat #13- 0112-82/Clone M1/70; eBioscience) antibodies for 15 min at 4°C. The cells were then incubated with anti-biotin magnetic beads for 20 min at 4°C and subsequently passed over an MS column. The cells were more than 90% Gr1^+^CD11b^+^ positive as determined by flow cytometry.

To isolate CD33^+^CD11b^+^HLA-DR^−^ cells from peripheral blood, PBMCs were first isolated by gradients of Histopage-1077 and Histopaque-1119 per manufacturer's instructions (Sigma-Aldrich, Saint Louis, MO), and then depleted of the HLA-DR^+^ cell subset using magnetic beads. The CD33^+^CD11b^+^ cell subset was then positively selected by incubating with biotin-coupled human anti-CD33 and anti-CD11b antibodies (Miltenyi Biotech) and isolated with anti-biotin magnetic beads.

Cells were cultured in RPMI-1640 medium (Invitrogen, Carlsbad, CA) supplemented with 100 U/ml penicillin, 100 μg/ml streptomycin, 2 mM L-glutamine (all from Hyclone Laboratories, Logan, UT), and 10% fetal bovine serum (Atlanta Biologicals, Lawrenceville, GA) at 37 °C and 5% CO_2_.

### Purification of exosomes

We assessed whether exosomes contain mediators that modify S100A9 protein localization in MDSCs. Exosomes were purified from blood plasma or MDSC culture supernatants.in cell culture, exosomes were purified using exoEasy Maxi Kit per manufacturer's protocol (Qiagen, Valencia, CA). For RNA expression analysis, exosomes were purified and RNA was isolated using exoRNeasy Starter Kit (Qiagen).

### Protein extracts

For whole cell lysates, the Gr1^+^CD11b^+^ cells were lysed in 1X RIPA buffer containing 50 mM Tris-HCl [pH 7.4], 150 mM NaCl, 1% NP-40, 0.25% sodium deoxycholic acid, and 1 mM EDTA (Millipore, Temecula, CA) plus 1X protease inhibitor cocktail. After 30 min on ice, cell lysates were cleared by centrifugation for 5 min at 4°C and 14,000 rpm. Protein concentrations were determined by Bradford assay (Bio-Rad), and aliquots were kept at −20°C.

Cytoplasmic and nuclear protein extracts were prepared using the NE-PER nuclear and cytoplasmic extraction kit (Pierce, Rockford, IL) per the manufacturer's instructions. Immediately after harvesting, the cells were washed in PBS and resuspended in CER1 lysis buffer with protease inhibitor cocktail and incubated on ice for 1 min. CER2 buffer was added, and the incubation continued for 5 min. Supernatants (cytoplasmic proteins) were removed by centrifugation for 5 min at 4°C and 14,000 rpm. The nuclear pellets were resuspended in NER lysis buffer with protease inhibitor cocktail and incubated for 40 min on ice with occasional vortexing. The nuclear proteins were recovered by centrifugation for 10 min at 4°C and 14,000 rpm. Protein aliquots were kept at −20°C.

### RNA immunoprecipitation

RNA immunoprecipitation was performed to detect Hotairm1 RNA binding to S100A9 protein. Briefly, Gr1^+^CD11b^+^ cells (^~^10 × 10^6^) were washed with warm PBS and incubated with 0.2% formaldehyde (in PBS) for 10 min at room temperature, to preserve RNA-protein complexes. Cross-linked cells were incubated for 30 min on ice in lysis buffer containing: 250 mM sucrose, 10 mM Tris-HCl [pH 7.5], 25 mM KCl, 5 mM MgCl2, 2 mM DTT, 0.5% NP-40, 0.5% deoxycholate, 30 U/ml RNase inhibitor, and 1x protease inhibitor cocktail. After DNase I treatment for 10 min at 37°C, cell lysates were cleared by centrifugation at 10,000 rpm for 10 min at 4°C. Cell lysates were immunoprecipitated with anti-S100A9 antibody (Cat #sc-58706; Santa Cruz Biotechnology, Dallas, TX) according to a published method [27]. Aliquots of S100A9-immunoprecipitated protein complexes were used for western blot or subjected to RNA isolation using TRIzol reagent (Invitrogen, Carlsbad, CA) and measurement of Hotairm1 levels by PCR.

### Western blot

Protein extracts or immunoprecipitated protein complexes were resolved by electrophoresis using SDS-10% polyacrylamide gel (Bio-Rad, Hercules, CA) and transferred to nitrocellulose membranes (Thermo Fisher Scientific, Waltham, MA). Membranes were blocked with 5% milk in Tris-buffered saline/Tween-20 for 1 hr at room temperature, and then probed overnight at 4°C with anti-S100A9 antibody (Cat #sc-58706; Santa Cruz Biotechnology). After washing, blots were incubated with the appropriate HRP-conjugated secondary antibody for 2 hr at room temperature. Proteins were detected with the enhanced chemiluminescence detection system (Thermo Fisher Scientific, Waltham, MA), the bands were visualized using the ChemiDoc XRS System (Bio-Rad), and the images were captured with the Image Lab Software V3.0. Membranes were stripped and reprobed with b-actin or nucleoporin antibody (Sant Cruz Biotechnology).

### Hotairm1 expression plasmid

Hotairm1 was cloned in pReceiver-M02 expression vector downstream of the CMV promoter. An empty control vector (pReceiver-M02CT) served as a negative control (GeneCopoeia, Rockville, MD).

### Cell transfection

For Hotairm1 expression, Hotairm1 plasmid was suspended in HiPerFect reagent at a 0.5 μg/ml final concentration (Qiagen, Valencia, CA). For Hotairm1 knockdown, Hotairm1-specific or scrambled siRNAs were suspended in HiPerFect reagent at a 0.5 μM final concentration. Gr1^+^CD11b^+^ cells were transfected according to the manufacturer's instructions and incubated with RPMI-1640 medium for 24-36 hr.

### Real-time PCR

Real-time qPCR (RT-qPCR) was used to determine the levels of Hotairm1 in exosomes, Gr1^+^CD11b^+^ cells, or S100A9 immunoprecipitates. Exosomes were purified from plasma or cell culture supernatants, and exosomal RNA was isolated using exoRNeasy Starter Kit (Qiagen). Total cellular RNA was isolated from Gr1^+^CD11b^+^ cells using TRIzol reagent (Invitrogen). Levels of Hotairm1 expression were measured using QuantiTect PCR Mastermix and RT2 lncRNAqPCR Assay primers (Qiagen). The expression level was calculated using the 2^−ΔΔCt^ cycle threshold method. Values were normalized to GAPDH RNA for total RNA or 18S RNA for exosomal RNA, amplified with QuantiTect Primer Assays. The PCR was performed in duplicate.

### T cells proliferation assay

We used a co-culture system to test the effects of MDSC-derived exosomes or MDSCs lacking Hotairm1 on spleen CD4 T cell proliferation and activation. Splenocytes were isolated from naive wild-type mice. CD4^+^ T cells were purified from splenocytes by positive selection using biotinylated anti-CD4 magnetic beads (Myltenyi) and labeled with carboxyfluoresceindiacetate, succinimidyl ester (CFSE) dye using the Vybrant CFDA SE Cell Tracer Kit (Invitrogen Molecular Probes, Eugene, OR). The cells were incubated for 10 min at room temperature with 10 μM CFSE dye. Gr1^+^CD11b^+^ cells were isolated from the bone marrow of sham and septic mice and cultured for 24 hr in serum-free medium, and exosomes were purified from the culture supernatants as described above. The labeled CD4^+^ cells were cultured in 12-well plates with naive Gr1^+^CD11b^+^ cells in the presence of exosomes (50 μg/ well). In other experiments, CD4^+^ T cells were co-cultured in a 1:1 ratio with late sepsis Gr1^+^CD11b^+^ cells in which Hotairm1 was first knocked down for 36 hr with a pool of siRNAs. Anti-CD3 antibody plus anti-CD28 antibody (1 μg/ml each) were added to the cultures to stimulate T cell activation and proliferation. After 3 days, the culture supernatants were collected and used to measure IFN-ϒ levels by ELISA. The cells were harvested and CD4^+^ T cell proliferation was determined by the stepwise dilution of CFSE dye in dividing CD4^+^ T cells using flow cytometry.

### Arginase assay

Arginase 1 activity in Gr1^+^CD11b^+^cells was determined by measuring urea concentration (a by- product of arginase 1 activity) in the cell lysates using an arginase assay kit (Abnova, Walnut, CA) as described previously [28]. One unit of arginase 1 converts 1 μmol of L-arginine to ornithine and urea per minute at pH 9.5 and 37°C.

### ELISA

Enzyme-linked immunosorbent assay (ELISA) kits were used to measure the levels of S100A9 (MyBioSource, San Diego, CA) and IFNϒ (eBioscience) in cell culture supernatants. Samples were run in duplicate.

### Statistical analysis

Data were analyzed with Microsoft Excel, V3.0. Data are expressed as mean ± SD. Differences among groups were analyzed by a two-tailed student's t-test for two groups and by one-way ANOVA for multiple groups. Statistical significance with p-values <0.05 are reported.

## RESULTS

### MDSC-derived exosomes contain mediators that inhibit S100A9 secretion

Exosomes are nano-sized (30-100 nm) vesicles that are released to the extracellular space when fused with the plasma membrane [29,30]. Exosomes facilitate the direct transfer of their cargos of proteins, non-coding RNA and DNA between parent cell and recipient cell *in vivo* and *in vitro* [31,32]. We have shown that inhibition of S100A9 secretion and shuttling to the nucleus in Gr1^+^CD11b^+^ cells during late sepsis is associated with the development of MDSCs [13]. Because exosomes contents share some of the transcriptomic and proteomic signature of the parent cell [31,33], we wondered whether exosomes shed from late sepsis Gr1^+^CD11b^+^ MDSCs could be used to identify mediators that promote S100A9 nuclear localization.

**Figure 1:**
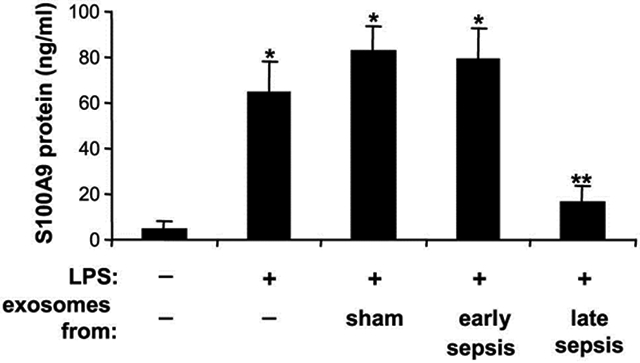
Exosomes shed from late sepsis MDSCs inhibit LPS-induced secretion of S100A9 protein from early sepsis MDSCs. Gr1^+^CD11b^+^cells were isolated from bone marrow of sham and septic mice by positive selection using anti-Gr1 antibody and magnetic beads. The cells were cultured for 24 hr in serum-free media. Culture supernatants were harvested, and exosomes were purified using exoEasy Maxi kit. Early sepsis Gr1^+^CD11b^+^ cells were cultured in 12-well plates with exosomes (50 μg/well) for 24 hr with or without 0.1 μg/ml of *E. coli* lipopolysaccharide (serotype 0111:B4; Sigma-Aldrich, St. Louis, MO). Levels of S100A9 protein in the culture super natants were measured by ELISA. Data are expressed as means ± SD of 6-8 mice (6-8 cultures/group) from three experiments. *p/**p < 0.05, versus exosomes from sham or early sepsis.

To test this, we cultured Gr1^+^CD11b^+^ cells from bone marrow of early septic mice - where S100A9 resides in cytosol and is readily secreted [13] - with exosomes purified from cultured Gr1^+^CD11b^+^ cells isolated from sham or septic mice. Then, the cells were stimulated with gram-negative bacterial lipopolysaccharide (LPS) to induce S100A9 secretion. We used Gr1^+^CD11b^+^ cells from bone marrow of early septic mice because, unlike cells from late septic mice, they can secrete S100A9 protein following stimulation with LPS [13]. S100A9 secretion significantly increased following stimulation with LPS, and was increased further slightly in the presence of exosomes from sham or early sepsis Gr1^+^CD11b^+^ cells (Figure 1). Notably, exosomes derived from late sepsis Gr1^+^CD11b^+^ MDSCs decreased S100A9 secretion significantly. These results suggest that late sepsis MDSC-derived exosomes contain inhibitors of S100A9 secretion.

### Late sepsis MDSC-derived exosomes switch naive Gr1^+^CD11b^+^ cells into immunosuppressive Gr1^+^CD11b^+^ MDSCs

**Figure 2:**
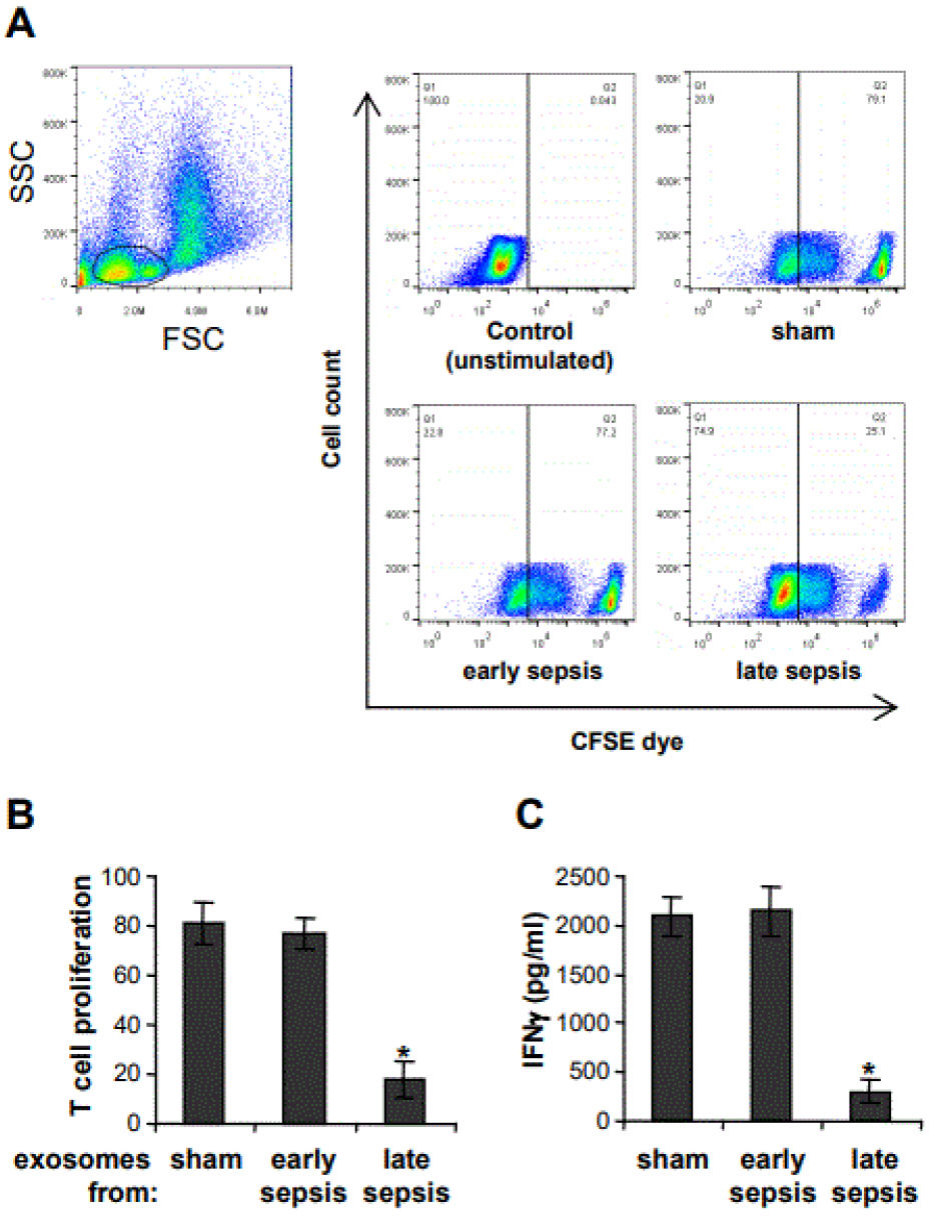
Late sepsis MDSC-derived exosomes switch naïve Gr1^+^CD11b^+^ cells into the immnosuppressive phenotype. Gr1^+^CD11b^+^ cells were isolated from the bone marrow of sham and septic mice by positive selection using anti-Gr1 antibody and magnetic beads. The cells were cultured for 24 hr in serum-free media, and exosomes were purified from the culture supernatants using exoEasy Maxi kit. CD4^+^ T cells were isolated from splenocytes of naive mice with anti-CD4 antibody and labeled with the fluorescent dye CFSE. The CD4^+^ T cells were cultured with naive Gr1^+^CD11b^+^ cells (1:1 ratio) in the presence of exosomes (50 μg/well) for 3 days, and anti-CD3 plus anti-CD28 antibodies (1 μg/ml each) were added to the culture to activate T cells. The cells were harvested, and T cell proliferation was determined by the step- wise dilution of CFSE dye in dividing CD4^+^ T cells using flow cytometry. (A) Representative dot plots of CFSE positive T cells gated on CD4 are shown. (B) Summary data of flow cytometry. (C) The culture supernatants were used to determine IFNϒ levels by ELISA. Data are expressed as means ± SD of 6-9 mice (6-9 cultures/group) from three experiments. *p < 0.05.

MDSCs from late septic mice suppress T cell activation and proliferation [13]. We investigated whether MDSC-derived exosomes can induce an immunosuppressive phenotype in naive Gr1^+^CD11b^+^ cells, which cannot suppress T cell [13]. Spleen CD4^+^ T cells were isolated from naive mice and cultured with naive Gr1^+^CD11b^+^ cells in the presence of exosomes derived from cultures of sham or sepsis Gr1^+^CD11b^+^ cells. The T cells were stimulated with anti-CD28 and anti-CD3 antibodies. Flow cytometry analysis showed that exosomes derived from early sepsis Gr1^+^CD11b^+^ cells did not significantly affect T cell proliferation, as compared to sham Gr1^+^CD11b^+^ cells (Figures 2A and 2B). In addition, these exosomes had no significant effect on T cell activation as determined by IFNϒ production (Figure 2C). Notably, exosomes from late sepsis Gr1^+^CD11b^+^ cells significantly inhibited both T cell proliferation and IFNϒ production, suggesting that they contain mediators that render naive Grl^+^CD11b^+^ cells immunosuppressive.

### Expression of lncRNA Hotairm1 is upregulated in late sepsis Gr1^+^CD11b^+^ MDSCs

**Figure 3:**
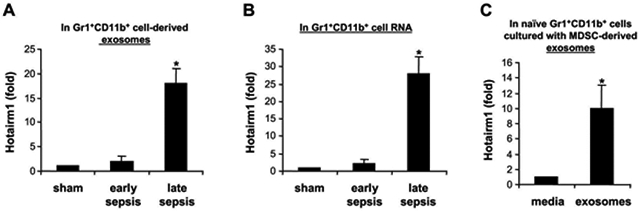
Hotairm1 expression is increased in late sepsis MDSCs. Gr1^+^CD11b^+^ cells were isolated from the bone marrow of sham and septic mice by positive selection using anti-Gr1 antibody and magnetic beads. (A) The cells were cultured for 24 hr in serum-free media. Exosomes were purified from the culture supernatants, and exosomal RNA was extracted with exoRNeasy Starter kit. Levels of Hotairm1 were determined by RT-qPCR using RT lncRNAqPCR Assay Primers (Qiagen). Values were normalized to 18S RNA. (B) Total RNA was isolated from Gr1^+^CD11b^+^ cells using TRIzol reagent, and levels of Hotaim1 were determined as in A. Values were normalized to GAPDH RNA. (C) Gr1^+^CD11b^+^ cells isolated from naïve mice were cultured for 24 hr without or with exosomes (50 μg/well), purified from late sepsis MDSC culture. The cells were harvested, total RNA was extracted and levels of Hotairm1 were determined as in B. PCR was performed in duplicate. Data are presented relative to sham or media control (1-fold). Data in A and B are expressed as means ± SD of 6-9 mice/group from three experiments. Data in C are expressed as means ± SD of 7 cultures from two experiments. *p < 0.05.

Re-localization of S100A9 protein from cytosol to nucleus of Gr1^+^CD11b^+^ cells occurs during late sepsis [13]. Because exosomes derived from late sepsis Gr1^+^CD11b^+^ MDSCs blocked S100A9 secretion and switched naive Gr1^+^CD11b^+^ cells to MDSCs (Figures 1 and 2); and because most immune-related lncRNAs can bind to and affect protein modifications and transport [21,22,24], we hypothesized that lncRNAs in exosomes contribute to S100A9 nuclear localization in Gr1^+^CD11b^+^ cells after early sepsis response. To test this, we profiled lncRNA expression in exosomes derived from sham and sepsis Gr1^+^CD11b^+^ cells, using mouse lncRNA expression Array V3.0 (Arraystar; Cat #AS-S-LNC-M). Sixteen lncRNAs simultaneously increased or decreased by ~2- to 4-fold in early and late sepsis Gr1^+^CD11b^+^ cells. Notably, lncRNA Hotairm1 (HOXA transcript antisense RNA, myeloid-specific 1) markedly increased in Gr1^+^CD11b^+^ cells after early sepsis. Using qPCR, we observed an 18-fold increase in Hotairm1 in exosomes from late sepsis Gr1^+^CD11b^+^ cells versus 2-fold in early sepsis cells (Figure 3A). A 29-fold increase in Hotairm1 transcripts occurred in total RNA extracted from late sepsis Gr1^+^CD11b^+^ cells (Figure 3B) and a 13-fold increase in plasma exosomes from late septic mice (data not shown). In addition, Hotairm1 was detected at high levels in naive Gr1^+^CD11b^+^ cells after culturing with exosomes derived from late sepsis Gr1^+^CD11b^+^ cells (Figure 3C).

### Knockdown of Hotairm1 attenuates immunosuppressive functions of late sepsis Gr1^+^CD11b^+^ MDSCs

**Figure 4:**
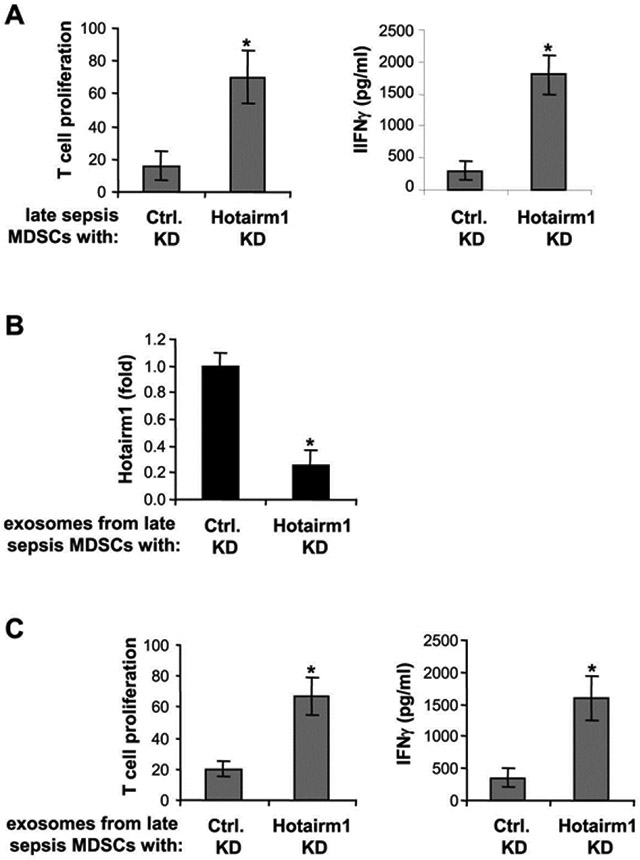
Knockdown of Hotairm1 in late sepsis MDSCs attenuates their immunosuppressive functions. Gr1^+^CD11b^+^ cells were isolated from the bone marrow of late septic mice by positive selection using anti-Gr1 antibody and magnetic beads. The cells were transfected with Hotairm1-specific or scramble siRNAs for 36 hr. (A) Effects of MDSCs on T cell proliferation and activation. CD4^+^ T cells were isolated from splenocytes of naive mice with anti-CD4 antibody and labeled with the fluorescent dye CFSE. The late sepsis Gr1^+^CD11b^+^ cells with Hotairm1 knockdown were then co-cultured (1:1 ratio). T cells were stimulated with anti-CD3 plus anti-CD28 antibodies (1 μg/ml each). After 3 days, the cells were harvested, and T cell proliferation and IFNϒ production were determined as in Figure 2. (B and C) Effect of exosomes lacking Hotairm1 on T cells. Late sepsis Gr1^+^CD11b^+^ cells with Hotairm1 knockdown were cultured for 24 hr in serum-free media. Culture supernatants were harvested, and exosomes were purified using exoEasy Maxi kit. (B) Levels of Hotairm1 in exosomal RNA was determined by RT-qPCR. Values were normalized to 18S RNA. (C) Spleen CD4^+^ T cells were labeled and cultured with naive Gr1^+^CD11b^+^ cells (as described in Figure 2) in the presence of Hotairm1-lacking exosomes. T cell proliferation and IFNϒ production were measured as in A. Data are expressed as means ± SD of 5-6 mice/group from three experiments. *p < 0.05.KD, knockdown.

We then asked the question whether Hotairm1 knockdown affects Gr1^+^CD11b^+^ MDSCs suppressive functions. Late sepsis Gr1^+^CD11b^+^ cells with Hotairm1 knockdown were co-cultured with CD4^+^ T cells isolated from spleens of naive mice. T cell suppression assay showed that Hotairm1 knockdown significantly reduced the inhibitory effects of Gr1^+^CD11b^+^ MDSCs on CD4^+^ T cell proliferation (Figure 4A). In addition, T cell activation, as measured by IFNϒ production, increased significantly. Because Gr1^+^CD11b^+^ cells can take up purified exosomes (Figure 3C), we next examined immunosuppressive effects of exosomes lacking Hotairm1 on T cells. The CD4^+^ T cells were co-cultured with naive Gr1^+^CD11b^+^ cells, which cannot suppress T cell, in the presence of exosomes purified from cultures of late sepsis Gr1^+^CD11b^+^ MDSCs in which Hotairm1 was knocked down first. Real-time PCR showed that the knockdown significantly reduced Hotairm1 levels in the Gr1^+^CD11b^+^ MDSC-derived exosomes (Figure 4B). Importantly, these exosomes switched naive Gr1^+^CD11b^+^ cells into immunosuppressive cells, as demonstrated by the inhibition of T cell proliferation and IFNϒ production, which decreased significantly after Hotairm1 knockdown (Figure 4C). These results support that Hotairm1 promotes the suppressive functions of late sepsis Gr1^+^CD11b^+^ MDSCs.

### Hotairm1 shuttles S100A9 protein to the nucleus in late sepsis Gr1^+^CD11b^+^ MDSCs

**Figure 5:**
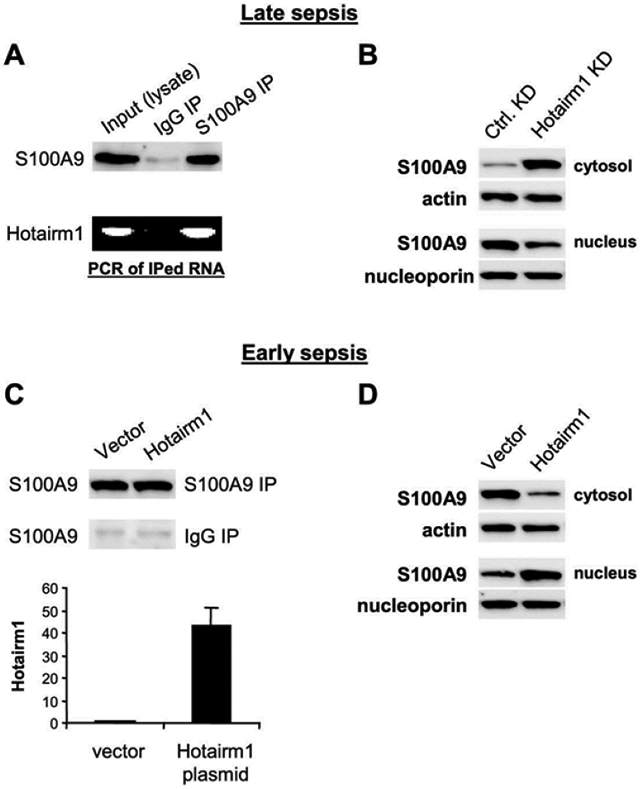
Hotairm1 binds to S100A9 in late sepsis MDSCs. Gr1^+^CD11b^+^ cells were isolated from the bone marrow of late septic mice using anti-Gr1 antibody and magnetic beads. (A) The cells (pooled from 2 mice) were treated with formaldehyde for reversible cross-linking of RNA-protein complexes. Cell lysates were prepared and immunoprecipitated with S100A9 or IgG antibody, and S100A9 levels were determined by western blot. The cross-linked RNA was extracted from the immunoprecipitated complexes using TRIzol reagent, and Hotairm1 levels were determined by standard PCR. (B) Hotairm1 knockdown relocalizes S100A9 in the cytosol. Late sepsis Gr1^+^CD11b^+^ cells were transfected with Hotairm1-specific or scramble siRNAs for 36 hr. Cytoplasmic and nuclear proteins were extracted, and levels of S100A9 were determined by Western blot. (C) Ectopic expression of Hotairm1 in early sepsis Gr1^+^CD11b^+^ cells moves S100A9 to the nucleus. The cells were transfected with Hotairm1 plasmid or a control vector for 24 hr. Cell lysates were prepared and immunoprecipitated with S100A9 or IgG antibody, and S100A9 levels were determined by Western blot. RNA was extracted from S100A9 IP and Hotairm1 levels were determined by RT-qPCR. Values were normalized to IgG IP samples and presented relative to vector. (D) Protein extracts were prepared, and levels of S100A9 were determined by Western blot. The results are representative of three experiments.

Most immune-related lncRNAs function through interactions with proteins [22,34]. To test whether Hotairm1 can bind S100A9 protein, we performed RNA-immunoprecipitation with cell lysates from late sepsis Gr1^+^CD11b^+^ cells. As shown in Figure 5A, PCR of cross-linked RNA extracted from S100A9 immunoprecipitates showed that Hotairm1 binds to S100A9 protein and transfers it from cytosol to nucleus in late sepsis Gr1^+^CD11b^+^ cells [13]. We tested the possibility that Hotairm1 shuttles S100A9 to the nucleus using western blot analysis, and found S100A9 moved from the nucleus to the cytosol after Hotairm1 knockdown (Figure 5B). Next, we investigated Hotairm1-S100A9 interactions using early sepsis Gr1^+^CD11b^+^ cells, where S100A9 protein resides mainly in the cytosol [13]. The cells were transfected with Hotairm1 expression plasmid. Immunoblotting of S100A9 IP showed that Hotairm1 plasmid transfection did not change S100A9 protein levels (Figure 5C). RNA-immunoprecipitation showed that Hotairm1 binding to S100A9 increased significantly after Hotairm1 plasmid transfection (Figure 5C). Notably, Hotairm1 expression resulted in S100A9 protein shuttling from the cytosol to the nucleus (Figure 5D). Together, these results indicate that Hotairm1 mediates S100A9 protein localization in the nucleus in Gr1^+^CD11b^+^ MDSCs in late sepsis.

### Increased Hotairm1 expression in early sepsis Gr1^+^CD11b^+^ MDSCs promotes an immunosuppressive phenotype

**Figure 6:**
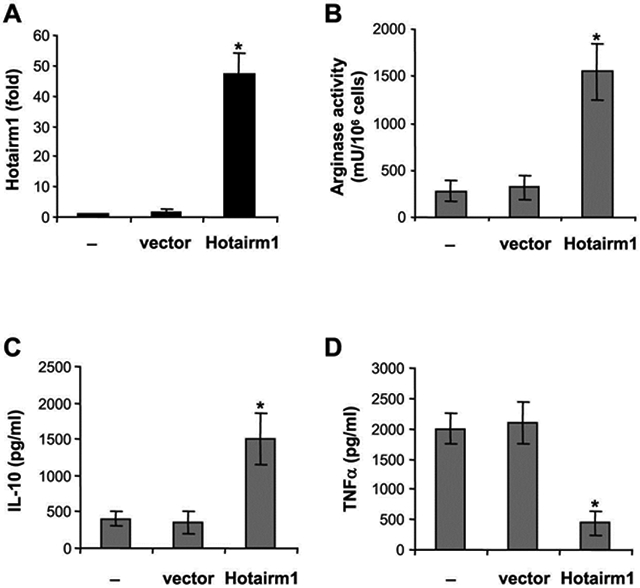
Overexpression of Hotairm1 in early sepsis Gr1^+^CD11b^+^ cells switches them to the immunosuppressive phenotype. Gr1^+^CD11b^+^ cells were isolated from the bone marrow of early septic mice using anti-Gr1 antibody and magnetic beads. The cells were transfected with Hotairm1 expression plasmid or an empty vector and cultured with 10 ng/ml of recombinant mouse IL-10. (A) After 24 hr, a portion of the cells was used for Hotairm1 expression measurement by RT-qPCR. The remainder of the cells were washed and stimulated with 1 μg/ml of of *E. coli* lipopolysaccharide (serotype 0111:B4; Sigma) for 24 hr. (B) The cells were harvested, lysed and analyzed for arginase activity. (C and D) Levels of IL-10 and TNFα in the culture supernatants were measured by ELISA. Data are expressed as means ± SD of 5-6 mice (5-6 cultures/group) from two experiments, *p < 0.05.

Knockdown of Hotairm1 in late sepsis Gr1^+^CD11b^+^ MDSCs attenuated their suppressive effects on CD4^+^ T cells (Figure 4). Gr1^+^CD11b^+^ MDSCs suppress T cells through the production of immunosuppressive mediators such as Arginase 1 and IL-10 [4]. We tested whether increasing Hotairm1 levels in early sepsis Gr1^+^CD11b^+^ MDSCs could affect Arginase 1 and IL-10 expressions. Gr1^+^CD11b^+^ cells from early septic mice were transfected with Hotairm1 expression plasmid, incubated with recombinant IL-10, and then stimulated with bacterial LPS. Hotairm1 expression was assessed by qPCR (Figure 6A). Importantly, Hotairm1 upregulation significantly increased Arginase 1 expression and IL-10 production significantly compared with cells transfected with an empty vector (Figures 6B and 6C). In contrast, levels of the proinflammatory cytokine TNFα were decreased (Figure 6D). In addition, transfection of Hotairm1 expression plasmid into Gr1^+^CD11b^+^ cells from late septic mice had no significant effects on the production of these inflammatory mediators, indicating that further increasing Hotairm1 in late sepsis MDSCs does not impact their immunosuppressive phenotype (Supplementary Figure 1). These results suggest that Hotairm1 upregulation during late sepsis induces the immunosuppressive phenotype of Gr1^+^CD11b^+^ MDSCs.

### Hotairm1 expression correlates with S100A9 localization in human MDSCs during sepsis

**Figure 7:**
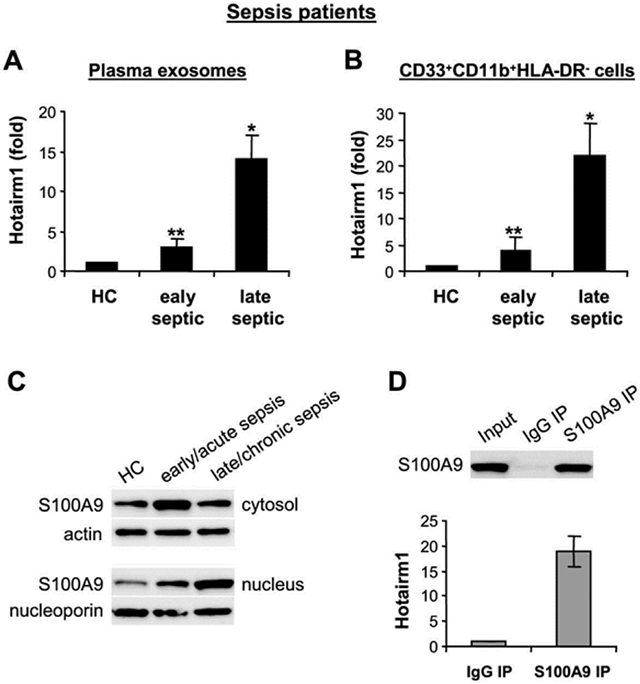
High levels of Hotairm1 in plasma exosomes and MDSCs from late septic patients. (A) Exosomes were purified from plasma and RNA was extracted using exoRNeasy Starter kit. (B) Peripheral blood CD33^+^CD11b+HLA-DR^−^ cells were isolated by magnetic cell separation. PBMCs were first purified and depleted of the HLA-DR^+^ cells. The HLA-DR^−^ cell population was then subjected to positive selection with biotin-coupled anti-CD33 antibody, followed by anti-CD11b antibody. Total RNA was extracted using TRIzol reagent. Levels of Hotairm1 were determined by RT-qPCR as in Figure 3. Values in A were normalized to 18S RNA, and values in B were normalized to GAPDH RNA. Data are expressed as means ± SD of 7-9 subjects/group and are presented relative to HC (1-fold), *p < 0.05, versus HC or early septic; **p < 0.05, versus late septic. HC, helthy control. (C) S100A9 accumulates in cytosol in CD33+CD11b+HLA-DR^−^ cells during late sepsis. Cytoplasmic and nuclear proteins were extracted from CD33^+^CD11b^+^HLA-DR^−^ cells, and levels of S100A9 were determined by Western blot. The results are representative of two Western blots. (D) Hotairm1 binds S100A9 protein during late sepsis. Cell lysates were prepared from CD33^+^CD11b^+^HLA-DR^−^ cells isolated from late septic patients (n=4) and immunoprecipitated with S100A9 or IgG antibody. S100A9 levels were determined by Western blot. RNA was extracted from S100A9 IP, and Hotairm1 levels were determined by RT-qPCR. Values were normalized to the input samples and are presented relative to IgG IP. The results are representative of two immunoprecipitations.

We have previously demonstrated that, similar to mice, S100A9 protein secretion is decreased significantly in late/chronic septic patients [13], pointing out to changes in S100A9 protein localization. We tested the possibility that Hotairm1 could modify S100A9 protein. First, we determined Hotairm1 levels in exosomes purified from patients plasma and in RNA from CD33^+^CD11b^+^HLA-DR^−^ MDSCs, the equivalent of mouse Gr1^+^CD11b^+^ MDSCs [7,35]. PCR analysis showed that Hotairm1 levels were significantly higher in plasma exosomes from late septic compared with early septic patients (Figure 7A). Hotairm1 levels were also significantly elevated in CD33^+^CD11b^+^HLA-DR^−^ MDSCs in late septic patients (Figure 7B). Notably, higher Hotairm1 expression in late septic patients correlated with S100A9 protein accumulation in the nucleus (Figure 7C). In addition, RNA-immunoprecipitation showed Hotairm1 binding to S100A9 protein in CD33^+^CD11b^+^HLA-DR^−^ MDSCs in late septic patients (Figure 7D). These results show that Hotairm1 promotes S100A9 protein accumulation in the nucleus in human MDSCs during chronic sepsis.

## DISCUSSION

The major finding of this study is that Hotairm1 dependent translocation of S100A9 protein from the cytosol to the nucleus in Gr1^+^CD11b^+^ myeloid precursors predominates during the late/chronic stage of sepsis and supports the MDSC repressor function. Our previous study showed that genetic deletion of S100A9 in mice improved late sepsis survival [13]. Mechanistically, increased expression of Hotairm1 associates with its binding to and shuttling S100A9 protein from the cytosol to the nucleus in MDSCs after early sepsis response. Ectopic expression of Hotairm1 in Gr1^+^CD11b^+^ cells from early/acutely septic mice with cytosolic S100A9 [13] shuttled S100A9 to the nucleus. Notably and of translational value, we found Hotairm1 binding to S100A9 in CD33^+^CD11b^+^HLA-DR^−^ MDSCs from chronically septic patients. Together, this study is compatible with the new concept that Hotairm1 acts as a molecular chaperone to modify S100A9 subcellular localization and switch Gr1^+^CD11b^+^ myeloid precursors into Gr1^+^CD11b^+^ MDSCs. The exact mechanism by which Hotairm1 promotes late sepsis is unclear, but we predict that Hotairm1-driven and nuclear S100A9 acts as a transcription co-factor to induce expression of immunosuppressive genes in Gr1^+^CD11b^+^ myeloid precursors, thus contributing to the MDSC development and late sepsis phenotype.

S100A9 expression markedly increases during early/acute sepsis, but significantly decreases in late septic mice concurrently with its translocation to the nucleus in Gr1^+^CD11b^+^ MDSCs [13]. In this study we made the novel observation that exosomes shed from late sepsis Gr1^+^CD11b^+^ MDSCs inhibited the LPS-induced secretion of S100A9 from early sepsis Gr1^+^CD11b^+^ cells. Notably, these exosomes also promoted an immunosuppressive phenotype in Gr1^+^CD11b^+^ cells, as evidenced by suppressing T cell activation and proliferation; a main feature of MDSCs [36]. Although mouse MDSCs have similar phenotype (Gr1^+^CD11b^+^) throughout sepsis, Gr1^+^CD11b^+^ cells that expand during early/acute sepsis for up to 5 days are not immunosuppressive [4,25]. In contrast, and after day 6 in our model, sepsis Gr1^+^CD11b^+^ cells become immunosuppressive, with higher levels of S100A9 protein in the nucleus [13]. Importantly, we observed significant increases in Hotairm1 levels in late sepsis Gr1^+^CD11b^+^ cells as well as in exosomes derived from their culture compared with early sepsis Gr1^+^CD11b^+^ cells. In addition, late sepsis Gr1^+^CD11b^+^ MDSCs with Hotairm1 knockdown could not inhibit T cells activation and proliferation. These findings suggest that Hotairm1 plays a pivotal role in generating immunosuppressive Gr1^+^CD11b^+^ MDSCs after acute sepsis in mice and in established chronic sepsis in humans.

Hotairm1 is located at the 3' end of the HOXA cluster between HOXA1 and HOXA2 genes [37]. Limited studies on Hotairm1 show its expression is specific to the myeloid lineage. Hotairm1 is low in human normal myeloid progenitors, but significantly increases in more mature myeloid leukocytes, with highest levels in neutrophils. MDSCs are progenitors and precursors of monocytes, granulocytes and dendritic cells, and are considered intermediates in the myeloid differentiation program and unable to give rise to mature cells [2]. Thus, the elevated levels of Hotairm1 suggest that its expression is part of the immunopathological response to sepsis. In contrast, Hotairm1 expression is decreased in CD33^+^ MDSCs from patients with lung cancer [38]. In that study, the common myeloid marker CD33 identified MDSCs, which is not sufficient to phenotype human MDSCs [35,39]. In addition, Hotairm1 is significantly increased during retinoic acid-induced granulocytic differentiation of the NB4 human acute promyelocytic leukemia cell line [37]. Together, these data support that Hotairm1 expression pattern in immature myeloid cells depends on the underlying pathological conditions. In support of this, MDSC expansion and suppressive functions depend largely on the inflammatory microenvironment in which they arise [35,40].

## CONCLUSION

In summary, we identified a novel molecular pathway leading to MDSC expansion during late/chronic sepsis. We discovered that following its increased expression, lncRNA Hotairm1 binds to and transfers S100A9 protein from the cytosol to the nucleus, where it promotes the development of Gr1^+^CD11b^+^ MDSC repressor phenotype. Since genetic targeting of S100A9 in mice limits MDSC expansion and sepsis-associated immunosuppression, molecular targeting of Hotairm1 is a plausible drug target for reversing sepsis-induced chronic immunosuppression.

## Supplementary Material

Supplementary data
