# Supplementary material for: Long Non-Coding RNA Hotairm1 Promotes S100A9 Support of MDSC Expansion during Sepsis
Source: J Clin Cell Immunol. Author manuscript; Available in PMC 2020 Dec 16. (PMC7744002)
Supplement: Supplementary data [file NIHMS1640051-supplement-Supplementary_data.pdf]

Supplementary Figure 1

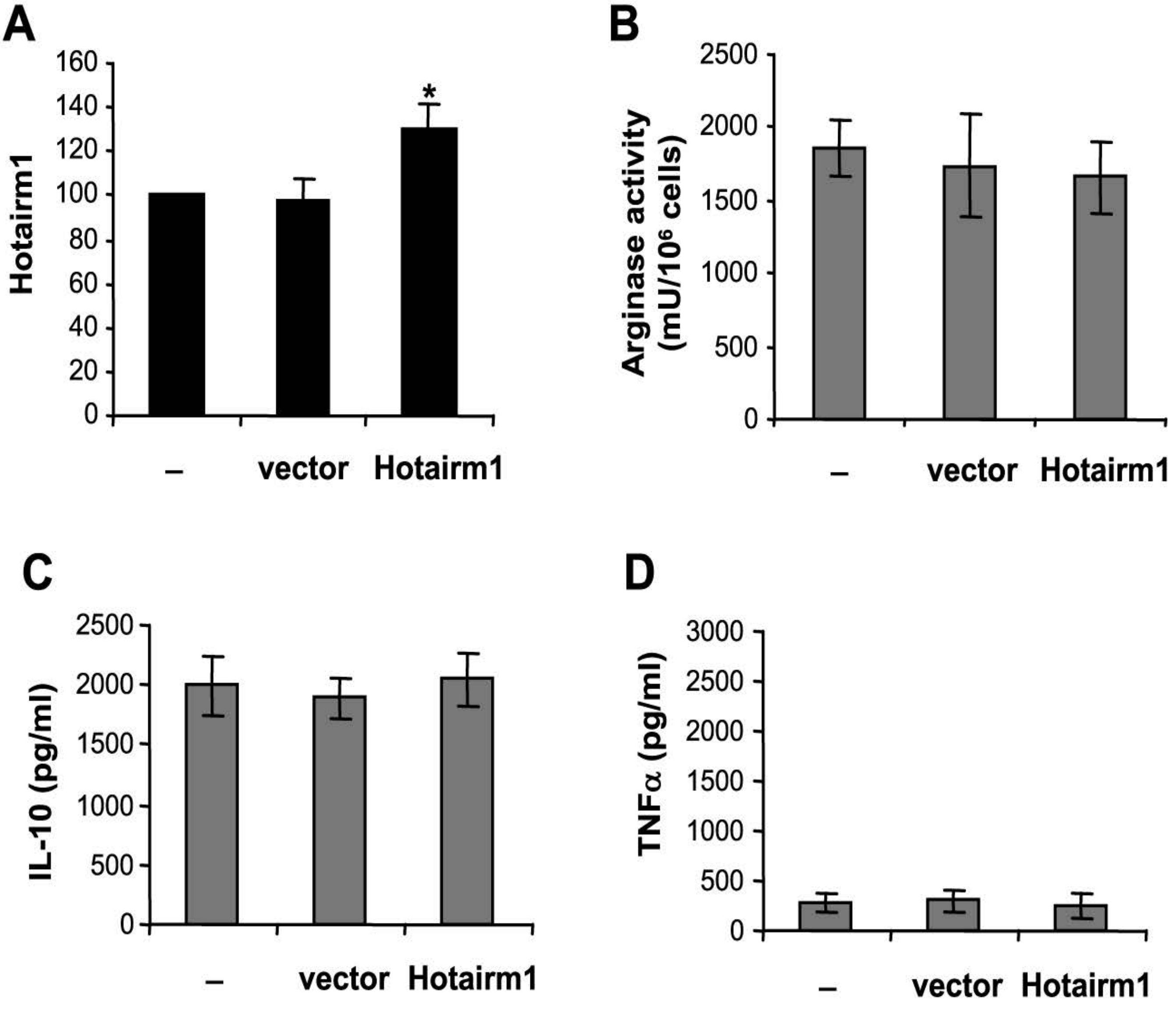

Supplementary Fig. 1. Production of inflammatory mediators by late sepsis Gr1$^+$CD11b$^+$ cells after transfection with Hotairm1 expression plasmid. Gr1$^+$CD11b$^+$ cells were isolated from the bone marrow of late septic mice. The cells were transfected with Hotairm1 expression plasmid. (**A**) After 24 hr, a portion of the cells was used to measure Hotairm1 level by RT-qPCR. Values were normalized to GAPDH RNA and are presented relative to cells without transfection (100 %). The cells were washed and stimulated with 1 μg/ml of bacterial lipopolysaccharide (LPS) for 24 hr. (**B**) The cells were harvested, and cell lysates were used for determining levels of arginase activity. (**C and D**) Levels of IL-10 and TNFα in the culture supernatants were measured by ELISA. Data are expressed as means ± SD of 5 mice (5 cultures/group). *$p < 0.05$.
